# Unlocking the synergistic potential of sensor technologies in grassland research

**DOI:** 10.1007/s44397-025-00020-2

**Published:** 2025-11-17

**Authors:** Keiji Jindo, Jouke Oenema, Yuta Miyoshi, Fedde Sijbrandij, Bernardo Maestrini, Idse Hoving, Hitoshi Nishikawa, Corne Kempenaar

**Affiliations:** 1 Agrosystems Research Group, Plant Science, Wageningen University & Research, Wageningen, 6700 AA The Netherlands; 2 Takasaki Institute for Advanced Quantum Science, National Institutes for Quantum Science and Technology (QST), Takasaki, 3701292 Japan; 3 Wageningen Plant Research, Wageningen University and Research, Wageningen, 6700 AA The Netherlands; 4 Animal Farming System, Wageningen Livestock Research, Wageningen University & Research, De Elst, 6708WD The Netherlands; 5 KUBOTA Holdings Europe B.V, Hoofdweg 1278, Nieuw-Vennep, 2153LR The Netherlands

**Keywords:** Precision agriculture, Digital platform, Remote sensing, Synthetic-aperture radar, Hyperspectral sensor, Multi-spectral sensors, Quantum sensors

## Abstract

In recent decades, the rapid advancement of sensor technologies has revolutionized research in grassland ecosystems. A wide array of sensor technologies has significantly enhanced field-based studies, contributing to a deeper understanding of grassland conditions. This review examines the synergistic potential of integrating remote sensing (RS) with various sensor technologies, such as hyperspectral, multispectral, thermal, and electrochemical sensors, highlighting their crucial role in understanding field conditions. The bibliometric analysis shows the chronological change in the usage and purposes of various RS technologies. Additionally, integrating these technologies into digital platforms offers holistic solutions for grassland management, benefiting farmers and researchers alike. A key contribution of this review is the organization of the grassland sensor landscape into four complementary pillars—structural, spectral, quantum, and proximal/physiological—and their integration with remote sensing, model–data assimilation, and digital platforms. We also synthesize emerging quantum-based approaches (e.g., cosmic-ray neutron sensing, neutron probes, graphene quantum-dot devices) and discuss their practical relevance for grassland monitoring.

**Supplementary Information:**

The online version contains supplementary material available at 10.1007/s44397-025-00020-2.

## Introduction

 Grassland ecosystems are critical for biodiversity, carbon storage, and livestock production. In Europe, grassland covers ~ 33% of agricultural land [1]. Yet they are highly vulnerable to climate change, including droughts, floods, and extreme events. Species diversity can improve resilience [2, 3]. Common bentgrass (*Agrostis capillaris*) and Sheep Fescue (*Festuca ovina*) are well-known resistant species in European grassland [4, 5], while Prairie clover (*Dalea spp.*) and Buffalo grass (*Buchloë dactyloides*) serve similar roles in the United States [6–9]. However, detecting and mapping such diversity with existing tools remains a challenge.

Grassland stores up to 25–34% of soil carbon stock worldwide [10]. Decomposition rates, especially under frequent cutting, can increase vulnerability to drought [11]. Yet, estimating cutting frequency and its effects is still time-consuming with current methods.

To meet these monitoring challenges, sensor technologies now span satellite- and unmanned aerial vehicle (UAV)-based optical and synthetic aperture radar (SAR), light detection and ranging (LiDAR), proximal devices, and emerging quantum-based sensors. Recent advances allow finer spatial detail, higher temporal resolution, and broader biophysical insights.

Previous reviews have emphasized remote sensing (RS) or single sensor classes [12–14]. Less attention has been given to: (i) a unified framework that groups sensor families by measurement principle; (ii) strategies for combining modalities across plot, field, and landscape scales; and (iii) integration with grass and forage growth models through data assimilation (DA) and digital platforms for decision support.

This review:

1. proposes a four-pillar taxonomy—structural sensors (e.g., LiDAR, global navigation satellite system interferometric reflectometry, GNSS-IR); spectral sensors (multispectral, hyperspectral, near-infrared spectroscopy, NIRS, sun-induced fluorescence, SIF); quantum sensor technologies (e.g., neutron probes, cosmic-ray neutron sensing, CRNS, graphene quantum-dot prototypes); and proximal/physiological sensors (thermal, electrochemical, ultrasonic, leaf area index, LAI, analyzers);
2. presents a bibliometric trend analysis (2012–2025) highlighting the shift from satellite-centric to multi-sensor, machine learning (ML)-enabled monitoring; and.
3. synthesizes integration pathways linking sensors with grassland growth models and digital platforms.

We emphasize the synergistic use of sensor modalities (e.g., SAR with optical data for mowing detection, spectral with ultrasonic for biomass, cosmic-ray neutron sensing with remote sensing for soil–canopy water dynamics) and highlight field-scale applications relevant to both research and practice. The paper is structured as follows: Sect. 1 reviews the bibliometric analysis (2012–2025) and places it in historical perspective, tracing sensor use from the 1970s to the present. Sections 2–5 examine the four sensor groups, Sect. 6 discusses model–data assimilation, Sect. 7 surveys digital platforms, and the Conclusion outlines integration opportunities and future directions.

## Bibliometric analysis

### Methodology of a bibliometric study

This review adopts a semi-systematic approach supported by a bibliometric trend analysis. Unlike a fully systematic review, the aim here was not to exhaustively synthesize all existing studies, but rather to provide a structured and critical overview of sensor technologies in grassland and pasture research. A semi-systematic strategy was chosen because it allows us to trace the historical development of sensor applications, categorize technologies into thematic groups, and highlight emerging directions, while retaining flexibility to include studies that are particularly informative for the field.

To complement the qualitative synthesis, a bibliometric analysis was conducted using VOSviewer to visualize research trends over time and thematic linkages. The bibliometric mapping served to illustrate the growth and clustering of research activity rather than to determine study inclusion.

The literature search was conducted in Scopus using the following query:

(TITLE-ABS-KEY(sensor) AND TITLE-ABS-KEY(grass* OR pasture OR meadow OR turf)) AND ( LIMIT-TO ( LANGUAGE,*“English” ) ) AND ( LIMIT-TO ( SRCTYPE*,*“j” ) ) AND ( LIMIT-TO ( DOCTYPE*,*“ar” ) OR LIMIT-TO ( DOCTYPE*,*“re” ) ) AND PUBYEAR > 2012 AND PUBYEAR < 2025*.

**Fig. 1:**
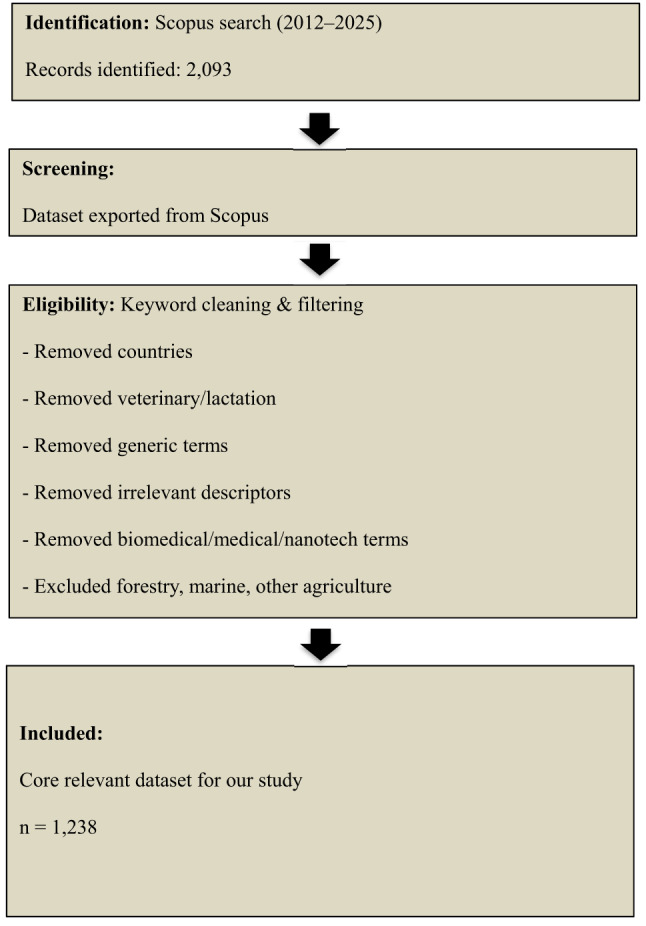
Chart flow of the semi-systematic review: identification (Scopus), screening, eligibility, and final inclusion

The search was executed on 1 September 2025 and limited to publications from 2012 onwards (the past 13 years). Eligible document types included peer-reviewed journal articles and reviews published in English. Index Keywords (controlled vocabulary) were used as the basis for the search strategy because they are more suitable for analyzing long-term historical trends. Unlike author-assigned keywords, index terms are standardized and consistently applied across years, ensuring comparability across different periods. This approach also reduces noise arising from variations in terminology, spelling, or phrasing, thereby providing a more reliable foundation for trend analysis. The query identified 2,093 records (Fig. 1). After duplicate removal and systematic keyword screening (removing countries, veterinary/animal terms, generic descriptors, biomedical/medical/nanotech terms, and non-core domains such as forestry or marine studies), 1,238 core relevant studies were retained for bibliometric analysis.

From this refined dataset, we selected more than 80 studies for detailed review. These were prioritized if they (i) contributed substantial empirical findings, (ii) represented diverse methodological or disciplinary approaches, and (iii) provided novel insights or critical perspectives.

In addition to the Scopus-based selection, we also incorporated emerging topics not fully captured by the query results, including quantum-based sensor technologies, crop growth models integrated with sensor applications, and digital platforms for decision-support services. These areas were included because they represent novel directions with high relevance to future grassland monitoring and management, even though they are not yet strongly represented in the indexed literature.

#### Result of bibliometric research for index keywords trend related to sensor technologies in grassland and pasture from 2012 to 2025

Across the dataset, “remote sensing” is the dominant keyword, underscoring its central role in grassland monitoring (Table 1). Within this category, satellite imagery—especially Landsat, MODIS, and Sentinel—appears most frequently, reflecting long-standing reliance on orbital data. By contrast, UAVs occur less often overall but mark a shift toward higher-resolution, site-specific monitoring [15–17]. Terms such as radiometers and antennas highlight ongoing interest in measurement precision and calibration. Multispectral and hyperspectral imaging are well represented, while thermal, infrared/near-infrared, radar, and LiDAR appear less frequently but signal growing interest in structural and soil moisture assessments.

**Fig. 2:**
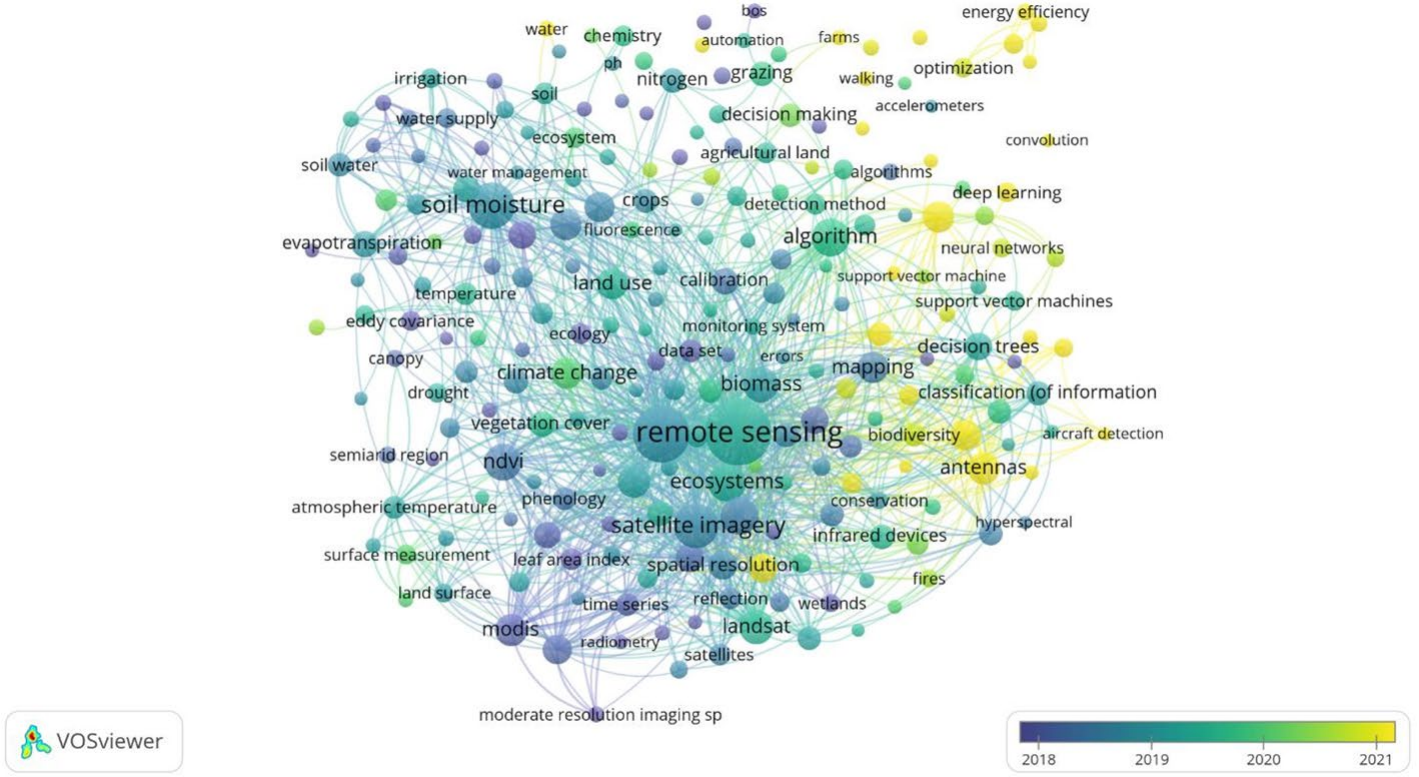
Bibliometric analysis of grassland monitoring research conducted with VOSviewer. The figure shows keyword co-occurrence networks, where node size reflects keyword frequency, link strength indicates co-occurrence relationships, and colors represent temporal clusters. This visualization highlights emerging trends and shifts in research focus over time

VOSviewer is a bibliometric tool that visualizes complex scholarly literature networks, helping researchers identify trends and relationships within academic publications [18]. Using VOSviewer, we traced keyword changes over 13 years (2012–2025) (see Supplementary Material for methods). From 2012 to 2016, satellite-based monitoring (Landsat, MODIS) dominated, with emphasis on vegetation indices (e.g., NDVI) and calibration methods (Fig. 2). In 2017, UAVs became more frequent, alongside growing use of hyperspectral and multispectral sensors. In 2021–2025, UAVs were often combined with optical, thermal, and radar sensors, while machine learning approaches (random forest (RF), support vector machines (SVM), deep learning) and digital platforms became increasingly popular.

**Table 1:** The main index keywords related to sensor technologies in grassland from 2013 to 2025 by using scopus

Sensor Technology (Keyword)	No of occurance	Main Application in Grassland Studies
Remote sensing	446	Umbrella term for satellite/UAV-based monitoring of vegetation and land cover
Satellite imagery / Satellite data*	310	Vegetation cover, biomass estimation, productivity mapping
Landsat	115	Long-term vegetation monitoring, land use/cover change
MODIS	84	Time-series vegetation indices (NDVI, productivity, drought monitoring)
Sentinel	64	Higher-resolution optical and radar data for vegetation and soil monitoring
UAV (Unmanned Aerial Vehicles)	76	High-resolution, site-specific monitoring of biomass, canopy traits, grazing impact
Radiometers	68	Ground-based measurement of spectral reflectance and energy balance
Antennas	86	Signal reception and transmission, microwave/radar applications
Hyperspectral imaging	53	Detailed vegetation traits, species composition, biochemical properties
Multispectral sensors	24	Vegetation indices (NDVI, EVI), canopy cover, biomass
Optical sensors	48	General optical reflectance, canopy structure and growth monitoring
Thermal remote sensing	29	Canopy temperature, water stress, evapotranspiration
Infrared / Near-infrared spectroscopy*	29	Vegetation biochemical properties, moisture content, chlorophyll
Radar / SAR*	60	Soil moisture estimation, structural mapping under cloud cover
GNSS	9	Positioning and reflectometry; limited use for land surface and soil moisture in grassland
LiDAR	30	3D canopy structure, biomass, grazing impact

#### Historical trend of the use of remote sensing data

**Fig. 3:**
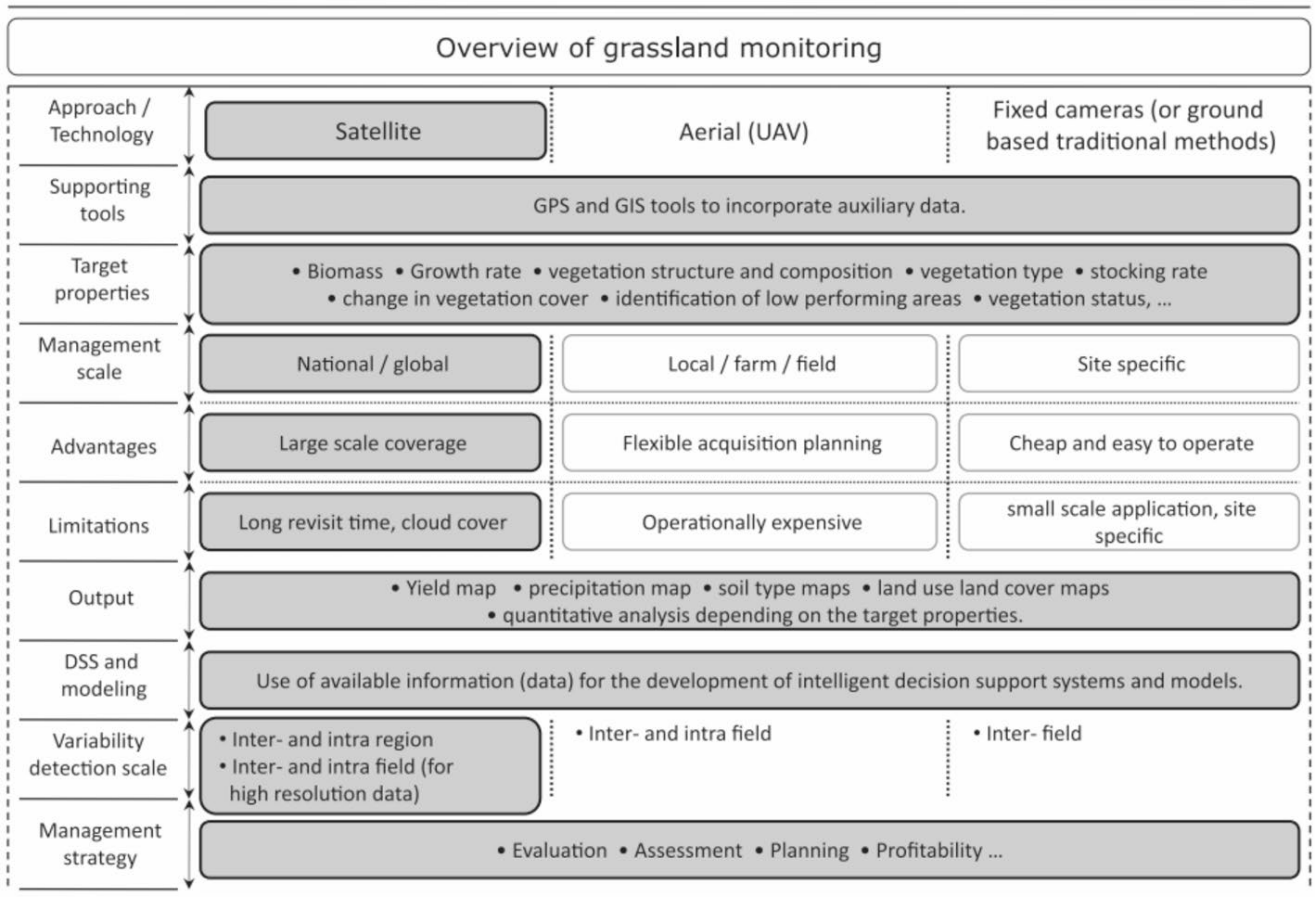
Overview of grassland monitoring using satellite, aerial (UAV), and fixed camera platforms. (adapted from Ali et al., [16]) [20]. The figure illustrates differences in spatial scales, advantages, limitations, outputs, and other remarks across these approaches

Although our bibliometric analysis (2012–2025) highlights that remote sensing and satellite imagery remain the most frequent and central keywords in grassland research, it is important to stress that the use of satellite data is not a recent development. In fact, satellite-based remote sensing was already applied in the early 1970s, for example through NASA’s ERTS mission (launched in 1972), to study correlations between spectral bands and above-ground biomass in grassland [19] This historical perspective shows that while our dataset reflects the last decade, the role of satellites has been foundational for grassland monitoring for more than five decades. Since then, RS technologies have been primarily used for two objectives in grassland research [20]: (1) monitoring plant status and estimating the yield and detecting potential plant stress and hazards in and around grassland, such as droughts, (2) classification:

1. There exist roughly two different types of using reflectance of remote sensing for monitoring plant status and estimating the yield: (a) Focusing on specific wavelengths and (b) using full spectral information across a wider range of wavelengths.

- Vegetative indices such as the normalized derivatives vegetation index (NDVI) and the enhanced vegetation index (EVI) were extensively used for monitoring [21]. Those indices are calculated by the wavelengths of near-infrared and green in an optical sensor. Also, fractional vegetation cover (FVC) is another well-known indicator for grassland monitoring [22]. Some researchers use meteorological and geographic data (e.g., altitude) to reinforce the estimation. Long-term grassland monitoring is crucial for maintaining ecosystem health [23]. One key indicator used in this monitoring is net primary productivity (NPP), which is defined as the rate of biomass accumulation per unit of ground surface area [23, 24]. NPP is an important indicator of forage availability. Typically, NPP is measured using photosynthetically active radiation. These satellite-based indicators are commonly used for detecting plant stress and natural hazards. As an example, the Global Grassland Drought Index (GDI) is an indicator, based on precipitation, soil moisture and canopy content using multiple data, including NDVI, NDII, LAI and LST [25]. Another report in Poland describes [26] Normalized Difference Drought Index (NDDI), calculated by the difference NDVI and Normalized Difference Water Index (NDWI), demonstrates distinct growth patterns in grassland across different areas in Poland in relation to the drought events. Satellite remote sensing methods have been widely used, while aerial and drone-based assessments are less common, to evaluate the effectiveness of indicators for early drought detection in grassland, probably due to the ability to capture large-scale, spatially explicit information about plant health and vigor [27].
- Radiative Transfer Models (RTM) utilize full spectral information across a wide range of wavelengths to simulate the interaction of radiation with matter, such as vegetation and soil. RTMs can estimate biophysical parameters (like Leaf Area Index, LAI) and reflectance values critical for understanding land surface characteristics and processes by considering various wavelengths. This approach allows for more accurate modeling and interpretation of remote sensing data in applications such as vegetation monitoring, climate studies, and environmental assessments [28].

(2) In grassland research, remote sensing data is utilized to create land use and cover maps. This is achieved by analyzing vegetation spectral signatures and employing image classification techniques, which are trained using ground truth data to differentiate between land types. Grassland includes various vegetation, like grasses, shrubs, and trees. Satellite imagery offers a synoptic view of grassland ecosystems and enables the classification of land use and cover types. Spectral indices, such as NDVI, differentiate vegetation based on leaf chlorophyll, which affects light reflectance in the electromagnetic spectrum. RS data is useful in grassland research for monitoring grassland loss [29]. It identifies restoration potential and quantifies grassland loss while tracking changes in distribution over time. RS data also monitors the effectiveness of restoration by comparing pre- and post-restoration imagery, as well as pre- and post-loss imagery. It assesses changes in vegetation, biodiversity, and ecosystem function, leading to adjustments in restoration strategies. Nowadays, classification algorithms, such as maximum likelihood or decision trees, are used to produce land use and cover maps of grassland ecosystems. These maps identify high biodiversity areas, quantify land use change, and monitor climate change impacts. Figure 3 represents the overview of grassland monitoring by Ali et al. [16]. This indicates that, at the time of their publication (2016), satellite data were still categorized at the national or global level. Also, the monitoring by a UAV device was considered an “operationally expensive” tool. The actual status of this information has changed these days. Additionally, UAV-based monitoring was previously considered operationally expensive. However, this has changed with advancements in technology and a reduction in costs [16].

#### Historical change in the use of RS data

Over the past few decades, the use of RS data in agriculture has progressed in both temporal and spatial resolution, expanding from landscape-level analysis to field-level analysis to the study of grazing animals. The improved temporal resolution of satellite imagery and the increased frequency of data availability are particularly important for grassland. In contrast to most open-field crops like maize, soybean, and wheat, grassland canopies are grazed or harvested continuously throughout the season, making timely imagery crucial for effective biomass monitoring. Additionally, advancements in the spatial resolution of satellite imagery are expanding grassland research from simply monitoring vegetation to detecting grazing animals. This is particularly useful information for managing extensive and remote beef production systems. For example, a report [29] detected the presence of cows in a Dutch grassland using very-high-resolution (VHR) satellite imagery. Additionally, UAVs can monitor herds, allowing for presence detection and animal counting [23, 24]. The rapid increase in temporal and spatial resolution of satellites will likely accelerate research in this field, increasing the possibility of coupling the monitoring of animals and vegetation.

Another significant advancement is the improvement in the resolution of optical sensors, which enables the measurement of a greater number of bands, including narrow bands. This results in the creation of numerous vegetation indices and enhancements in the detection of non-valid pixels, such as clouds and shadows. A third major development is the utilization of active sensors, such as Synthetic Aperture Radar (SAR), which provides cloud-independent measurements and results in increased image availability, or satellite sun-induced fluorescence, which can offer valuable insights into the efficiency of the photosynthetic process.

#### SAR data

The previously mentioned sensor technologies primarily rely on optical sensors. In contrast, the use of active sensors, such as Synthetic Aperture Radar (SAR), is also quite common. These sensors offer cloud-independent measurements, leading to enhanced consistency in image availability. Additionally, satellite sun-induced fluorescence can yield valuable insights into the efficiency of the photosynthetic process [25]. There are three different advantages of using SAR data:

1. Measuring soil moisture content by analyzing the backscatter of radar signs [28–30].
2. Measuring vegetation structure and biomass estimation by analyzing the backscatter data for grass height and density of vegetation. While optical data can provide information on vegetation phenology and chlorophyll content, SAR includes information on structure and biomass [31].
3. Detecting disturbances such as flood incidence by analyzing changes in backscatter over time [31–34].

Integrating radar and optical sensors has become a common approach in grassland research to address the limitations of relying on a single data source [31, 32]. This integration is primarily used for detecting mowed areas [33, 34] and estimating plant height [31]. Radar backscatter further enhances classification accuracy, especially in regions with high cloud cover [31].

The following sections present a range of sensor technologies applied in grassland research. We categorize them into four main groups according to their primary focus:

1. Structural mapping sensors such as Lidar and GNSS, which target plant shapes and canopy structures;
2. Spectral analysis sensors, including multispectral, hyperspectral, and near-infrared spectroscopy, which capture spectral characteristics of vegetation;
3. Quantum sensor technologies, such as neutron probe sensors and graphene quantum-dot devices, which represent emerging and innovative approaches;
4. Proximal physiological sensors, including thermal, electrochemical, ultrasonic, and LAI analyzers, which measure functional and physiological traits at the field scale.

The four-pillar classification—Structural Mapping, Spectral Analysis, Quantum, and Proximal Physiological Sensors—offers a comprehensive and functionally grounded framework for organizing sensor technologies in grassland research. Each category reflects a distinct measurement principle and ecological focus: structural sensors capture canopy geometry and terrain; spectral sensors decode biochemical and physiological traits; quantum sensors directly sense subsurface water dynamics; and proximal sensors provide in-situ diagnostics of plant and soil status. This taxonomy is both necessary and sufficient to span the full continuum of spatial scales (from leaf to landscape), sensing depths (surface to root zone), and data types (optical, thermal, electromagnetic, and nuclear), enabling coherent comparison and integration across modalities.

### Sensors focusing on plant shapes and canopy structures

#### LiDAR (light detection and ranging)

**Fig. 4:**
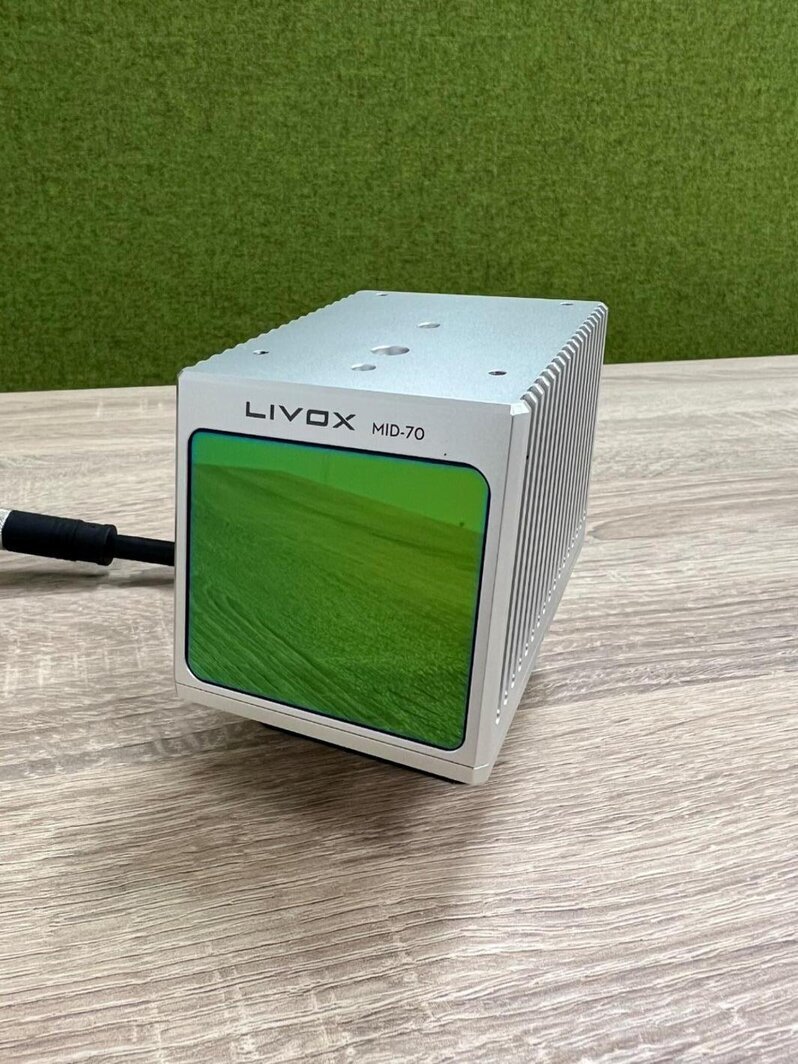
Low-cost LiDAR sensor, “MID-70”, from LIVOX Technology Company Limited

LiDAR technology is highly effective in grassland research, as it can create detailed digital elevation models to identify plant species based on vegetation height and structure. It can be used on UAVs to gather precise 3D data on vegetation, enabling the estimation of plant characteristics such as height, biomass, and canopy cover [35]. LiDAR data can be collected using airborne or ground-based systems. Airborne systems are ideal for larger studies, while ground-based systems offer high-resolution data for smaller-scale research. For instance, the MID-70 is an affordable LiDAR sensor with a wide 70.4-degree circular field of view (FOV) and a minimum measurement distance of 5 cm (Fig. 4). It can generate 3D point clouds, even in outdoor environments, and is now being considered for agricultural applications like crop sensing.

Despite its high spatial accuracy, LiDAR is subject to limitations such as vegetation occlusion, scan angle dependency, and sensitivity to surface reflectivity. In practical applications, it requires substantial post-processing and is affected by platform stability and weather conditions.

To address limitations related to scan angle distortion, platform instability, and vegetation occlusion, LiDAR systems can be integrated with inertial measurement units (IMUs) to improve georeferencing and motion compensation. This fusion enables more accurate point cloud generation, especially under dynamic flight conditions. Additionally, combining LiDAR with multispectral imagery or RGB cameras enhances canopy segmentation and biomass estimation. Machine learning techniques, such as CNN-based classifiers, can further refine object recognition and structural mapping.

The choice of LiDAR system depends on the research question and study area, with UAV-based systems ideal for small-scale studies and airborne or ground-based systems better suited for larger areas (Fig. 4).

#### Global navigation satellite system interferometric reflectometry (GNSS-IR)

Global Navigation Satellite System Interferometric Reflectometry (GNSS-IR) is a remote sensing technique that utilizes signals from GNSS satellites to measure surface reflections. It measures the phase delay and amplitude attenuation of the reflected signals to determine surface characteristics such as roughness and height. This technique is similar to radar altimetry but uses signals from GNSS satellites. GNSS-IR has applications in various fields, such as hydrology, geodesy, and oceanography, and it can be used to monitor changes in water levels, sea ice, and land surfaces. In grassland and pasture research, GNSS-IR can monitor and analyze changes in surface characteristics such as biomass, vegetation height, and soil moisture, which are important factors in pasture management and productivity [36].

There are several potential limitations to using GNSS-IR for grassland and pasture monitoring research. These include limited spatial resolution, making detecting small-scale changes in vegetation cover or soil moisture difficult. The quality of GNSS-IR data can also be affected by weather conditions such as clouds, rain, and snow, particularly in areas with frequent precipitation. Processing GNSS-IR data can be complex and requires specialized software and expertise, which can increase the cost and time needed for data analysis. Additionally, GNSS-IR requires specialized equipment, such as a GNSS receiver and an antenna, which can be expensive and may needaintenance or calibration [37].

To overcome limitations in spatial resolution and signal variability, GNSS-IR can be complemented by manual ground sampling to validate and calibrate reflectometry-derived estimates. This hybrid approach ensures higher confidence in soil moisture and vegetation height measurements, especially in heterogeneous landscapes. Additionally, co-location with other sensors such as TDR probes or multispectral imagers allows cross-validation and data fusion. Machine learning techniques can further support spatial interpolation and temporal smoothing of GNSS-IR signals. Integration with IMU (Inertial Measurement Unit) systems also improves antenna orientation tracking, enhancing signal stability and geolocation accuracy.

Despite these limitations, GNSS-IR remains a rational choice for long-term, low-maintenance monitoring of soil moisture and vegetation dynamics over large spatial scales. Its passive sensing nature and compatibility with existing GNSS infrastructure make it particularly suitable for semi-arid grasslands, rangelands, and remote areas where deploying dense sensor networks is impractical.

### Sensors focusing on spectral characteristics

#### Hyperspectral sensor

Hyperspectral sensors have several advantages for grassland research. They can capture data in hundreds of narrow spectral bands, allowing for detailed characterization of the spectral signature of different vegetation types. This enables differentiation between similar vegetation types and identifies areas with high biodiversity. Hyperspectral sensors can capture the unique spectral reflectance patterns of different grassland species, enabling the distinction between species based on their combinations of chlorophyll, pigments, and structural features. The technology can detect invasive grass species that threaten the native grassland ecosystem. Hyperspectral data can also be used to estimate a variety of biophysical and biochemical parameters related to vegetation. These parameters provide information on vegetation health, productivity, stress, and monitoring changes over time, such as leaf area index (LAI), chlorophyll content, water content, biomass, and protein. Combining hyperspectral sensors with other sensors can improve biomass estimation in spatially heterogeneous grassland. Hyperspectral sensors can also identify specific diseases in grassland, such as *Silybum marianum* infection [38]. Hyperspectral sensors can be mounted on various platforms, including satellites, aircraft, and drones, enabling efficient data collection across different spatial and temporal scales. A recent study demonstrated the high performance of hyperspectral sensors on a tractor for estimating the height and nitrogen content of perennial ryegrass.

However, hyperspectral sensors have disadvantages, including large and complex data requiring significant processing time, high cost, sensitivity to atmospheric interference, limited spatial coverage, and infrequent temporal coverage. It is important to match the scale size with the objective of the study to monitor habitat heterogeneity in the grassland.

To address the challenges of data complexity, atmospheric sensitivity, and limited temporal coverage, hyperspectral analysis can benefit from both data-driven and knowledge-driven approaches. Machine learning algorithms such as support vector machines (SVMs), random forests (RFs), and convolutional neural networks (CNNs) are increasingly used to classify vegetation traits and detect stress patterns. However, incorporating prior knowledge about specific wavelength regions—such as chlorophyll absorption around 680 nm or water content sensitivity near 970 nm—can significantly improve model interpretability and reduce overfitting. This hybrid strategy enables more robust feature selection and enhances the physiological relevance of spectral models.

Furthermore, hyperspectral data can be effectively integrated into multimodal frameworks, combining structural (e.g., LiDAR), thermal, and biochemical sensors. For example, fusing hyperspectral reflectance with LiDAR-derived canopy height allows simultaneous estimation of biomass and species composition. Similarly, combining hyperspectral and thermal data supports evapotranspiration modeling and drought stress detection. These multimodal approaches not only improve prediction accuracy but also enable cross-validation across sensor modalities, enhancing reliability in heterogeneous grassland environments.

Hyperspectral sensors remain valuable for detailed vegetation characterization in grassland research at a fine spatial scale, and their utility can be further enhanced through integration with prior spectral knowledge, machine learning techniques, and multimodal sensor fusion. These approaches unlock new potential for robust trait detection, stress monitoring, and ecosystem modeling under complex field conditions.

#### Multispectral sensor

Multispectral sensors can be utilized in grassland research to obtain information on various aspects of vegetation, like plant biomass, chlorophyll amount, and plant species composition. These sensors calculate various vegetation indices by measuring light reflectance at multiple wavelengths. For decades, multispectral sensors have been used in grassland research, and this technology has undergone significant advancement with the development of new sensors and data analysis techniques. One study used a drone-mounted multispectral sensor to create a map of invasive plant species in a grassland ecosystem [39]. This study reported that the sensor accurately differentiated between various plant species and helped devise effective strategies for controlling invasive species. Another study utilized a handheld multispectral sensor to estimate the above-ground biomass of a grassland ecosystem [40]. The study concluded that the sensor could provide precise biomass estimates, enhancing management practices for grazing and forage production. The strengths of multispectral sensors in grassland research include their high resolution, ability to collect data rapidly over large areas, and detection of subtle changes in vegetation health. However, the susceptibility to interference from atmospheric conditions, particularly clouds and smoke, is the major weakness of multispectral sensors in grassland research.

Although multispectral sensors capture fewer bands than hyperspectral systems, their effectiveness can be significantly enhanced through strategic wavelength selection. By leveraging prior knowledge—such as vegetation indices (e.g., NDVI, EVI) and known absorption features—alongside machine learning-based feature selection, multispectral data can be tailored to specific ecological targets. Commonly used algorithms include machine learningmethods such as RF, SVM, RFE, and multivariate anlaysis such as Principal Component Analysis (PCA), which help identify the most informative bands for classification or regression tasks. Moreover, multispectral workflows are often built upon RGB image processing pipelines, making them highly adaptable and scalable for researchers transitioning from conventional imaging to spectral analysis. In addition, multispectral sensors are generally more affordable than hyperspectral systems, offering a cost-effective solution for many grassland applications without compromising essential analytical capabilities.

#### Near-infrared spectroscopy (NIRS)

NIRS, a non-destructive analytical technique, uses a broadband light source to illuminate a sample and measure the reflected or transmitted light. The resulting spectrum can be used to determine the sample’s chemical composition. NIRS was initially used for laboratory-based analysis of plant and soil samples.

However, nowadays, many studies are mounting NIRS sensors on UAVs for grassland research for several purposes: (1) to determine the chemical composition of pasture, including sugar and protein content; (2) to identify plant species; (3) to monitor soil and plant health.

1. Crude protein is important to guarantee the feed quality for the livestock. Using sensors of near-infrared spectroscopy with a large dataset is a very useful approach to estimating the proximity of the protein content of the feed [41–43]. Recently, tractor-mounted sensors have been applied to measure crude protein and neutral detergent fibre digestibility [44].
2. Identification of species: NIRS has been used to accurately identify various grassland species, including forage grasses, legumes, and non-grass species [45, 46].
3. Monitoring soil and plant health. NIRS can measure the absorption of near-infrared light by organic molecules in the soil or plant material, which can be used to predict the sample’s chemical composition. NIRS has several advantages over traditional soil and plant analysis methods, such as high speed, low cost, and minimal sample preparation. NIRS has been used to monitor various soil properties in grassland ecosystems, including soil organic matter, total nitrogen, and soil pH. For instance, it is used to predict soil organic matter content in grassland, and it was found that NIRS provided accurate predictions of soil organic matter content [47].

There are some limitations to the use of NIRS in grassland research. One limitation is the need for accurate calibration, as the accuracy of NIRS measurements can be affected by variations in sample composition and moisture content. NIRS also has limitations in terms of spatial resolution, as it typically measures the composition of a bulk sample rather than individual plants or leaves. Additionally, NIRS may not be effective for detecting small changes in nutrient content, such as those that might occur with changes in soil fertility or management practices.

To overcome limitations related to calibration sensitivity and sample heterogeneity, NIRS applications in grassland research increasingly rely on advanced data-driven approaches. Machine learning algorithms such as Partial Least Squares Regression (PLSR), Support Vector Machines (SVM), and Artificial Neural Networks (ANN) are commonly used to build predictive models for forage quality, species identification, and soil composition. These models help extract meaningful spectral features even under variable field conditions.

In addition, Transfer Matrix methods have proven effective for adapting calibration models across different instruments, environments, or sample types. By mathematically transforming spectral data from one domain to another, transfer matrices allow researchers to reuse existing calibration datasets, reducing the need for extensive re-sampling and re-calibration. This is particularly valuable when deploying NIRS sensors on UAVs or tractors, where environmental variability and sensor drift can otherwise compromise accuracy.

Combining NIRS with multispectral or RGB imaging also enhances spatial resolution and enables hybrid workflows for mapping chemical traits across heterogeneous grassland plots. These integrative strategies expand the utility of NIRS beyond laboratory settings, making it a practical tool for in-field diagnostics and precision pasture management.

### Quantum sensor technology

Quantum-based sensors complement structural (geometry/imaging) and spectral (radiance/emissivity) approaches by directly sensing hydrogen-bearing phases—most notably liquid water—rather than optical or geometric surrogates. This yields two main benefits: subsurface access (centimeter–decimeter depths) that captures root-zone wetting and storage dynamics often missed or delayed in surface-limited spectral data, and scale bridging from depth-resolved points (neutron probes) to field-average footprints (CRNS) and miniaturized, high-sensitivity local readouts (GQDs).

#### Neutron probe sensor

Neutron probes are a classic tool for measuring soil moisture based on neutron scattering. A typical probe contains a radioisotope source (e.g., Americium-241/Beryllium) that emits fast neutrons, which are strongly moderated (slowed) by hydrogen atoms in soil water [48]; the backscatter of slow (thermal) neutrons is counted by a detector and related to volumetric water content [49]. By lowering the probe into access tubes installed in the ground, each measurement integrates moisture over a relatively large volume of soil (roughly a 20–30 cm radius sphere around the probe) [50]. Neutron scattering has been used for decades in grassland and agricultural research to track soil moisture profiles over time [51]. Neutron probes act as point-scale, depth-resolved anchors that ground spectral/structural observations with absolute root-zone moisture profiles at the plot scale.

Neutron probe measurements are generally robust and well-calibrated, providing reliable in situ soil moisture data that is considered highly accurate compared to other techniques [50]. In most cases, the readings are unaffected by moderate soil salinity or texture variations [52]. This allows continuous monitoring of soil water dynamics (e.g., seasonal drying and rewetting under pastures) without disturbing the soil, which is crucial for irrigation management and understanding plant-available water [53]. Because the neutron method measures hydrogen content directly, factors like soil temperature or bulk electrical conductivity have minimal impact on the readings. Only under unusually saline conditions would calibration be significantly affected [52].

**Fig. 5:**
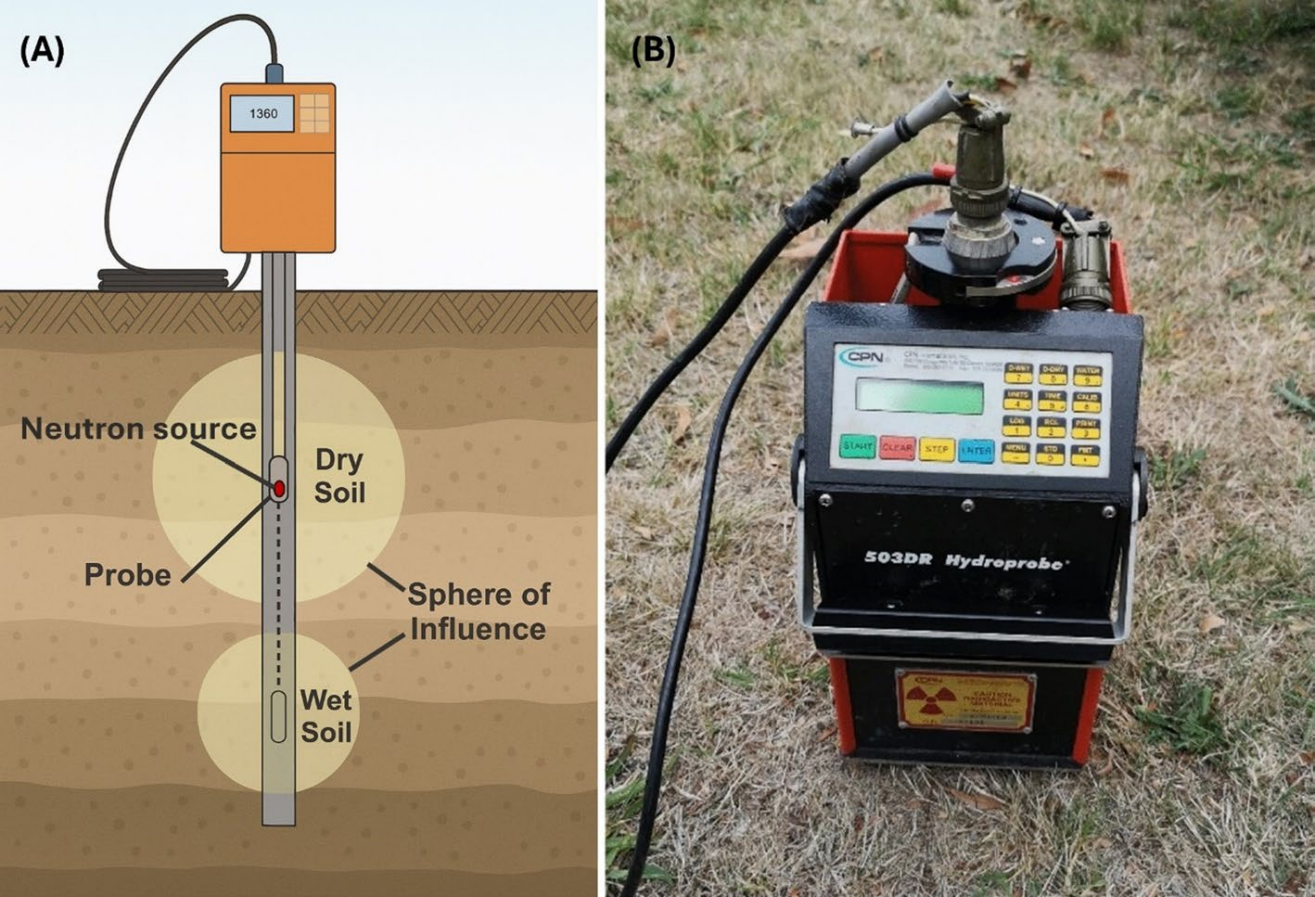
(A) Schematic illustration of a neutron probe setup for measuring soil moisture. (B) Photograph of a field-deployed neutron probe device (CPN 503DR Hydroprobe^®^) from Sun et al. (2023), Acta Geotechnica 18:5901–5919, DOI:10.1007/s11440-023-02075-0. Licensed under CC BY 4.0 [54]

Neutron probes can provide accurate in situ soil-water profiles once calibrated; however, a single neutron probe samples only a point location (the volume around one access tube), so multiple tubes are required to capture spatial moisture variability across a field. The method also involves handling a radioactive source, necessitating strict safety protocols and regulatory compliance [49]. There is reduced sensitivity near the soil surface – measurements within about the top 10 cm are less accurate due to neutron escape into the air, leading to underestimation of surface soil moisture (an effect most pronounced in wet soil) [51]. Furthermore, installing access tubes disturbs the soil initially, and the measurements are relatively labor-intensive (requiring manual readings at each tube). These practical constraints, along with the need for radioactive source licensing, can limit broader adoption and, in some contexts, have shifted attention toward newer sensing technologies that are non-radioactive and more easily automated. For example, modern electromagnetic sensors (such as TDR/FDR probes) and cosmic-ray neutron instruments can provide soil moisture data with less labor and no radioactive materials. Nevertheless, where licensing and staffing requirements can be met, neutron probes remain a robust option for in situ soil-water profiling. Near-term deployments commonly co-locate shallow electromagnetic probes (e.g., TDR/FDR) to bound near-surface underestimation, maintain site-specific calibration with routine standard counts and intercomparison checks, and codify licensing, training, and sealed-source handling in SOPs to reduce operational friction (Fig 5).

#### Cosmic-ray neutron sensing (CRNS)

**Fig. 6:**
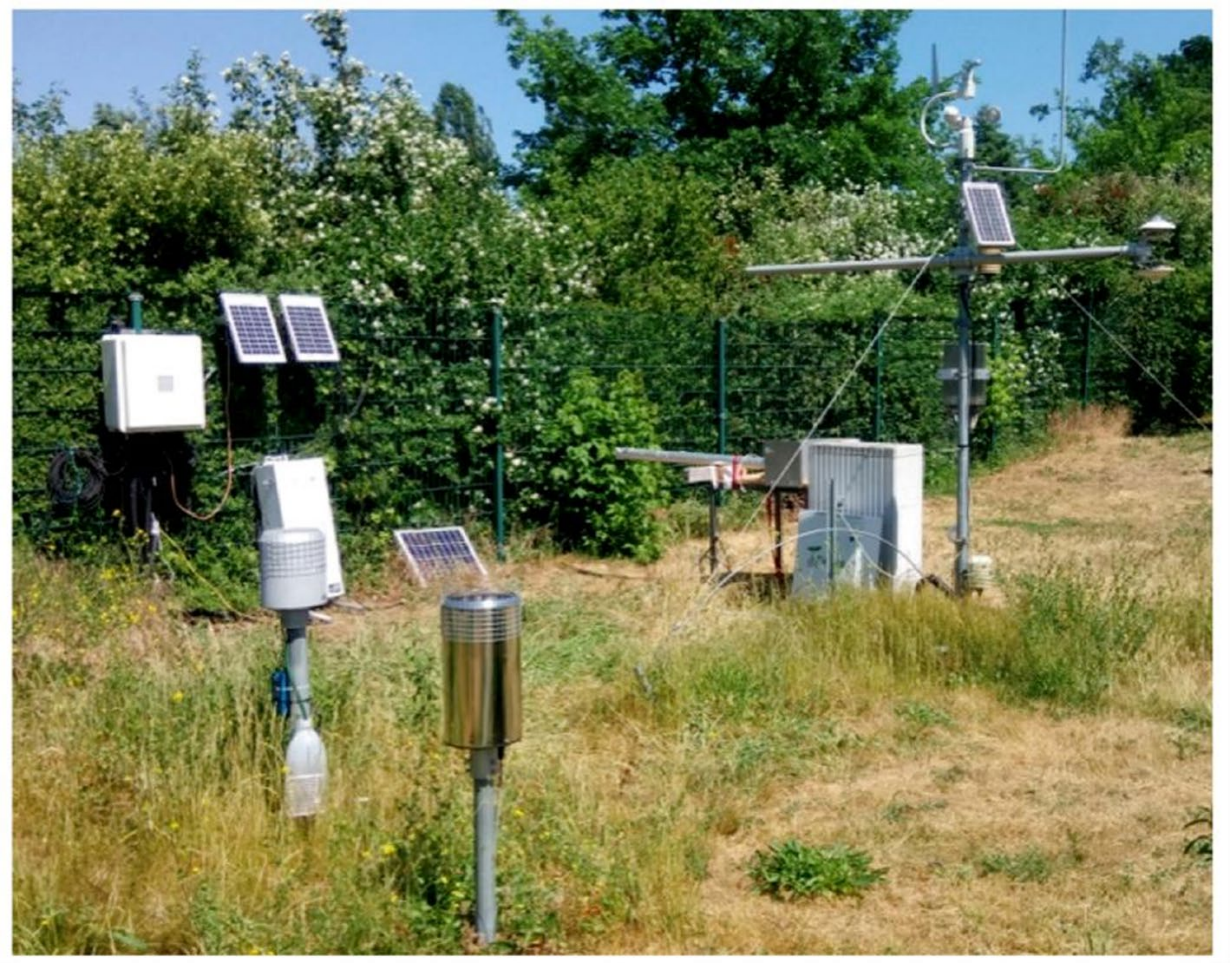
Picture of the installation of CRNS probes from Stevanato et al. (2019), *Agriculture*, 9:202, DOI: 10.3390/agriculture9090202. Licensed under CC BY 4.0 [61]

Cosmic-ray neutron sensing (CRNS) is a field-scale method for non-invasive soil moisture monitoring that utilizes ambient neutrons produced by cosmic-ray interactions in the atmosphere (Fig. 6). High-energy galactic cosmic rays initiate nuclear cascades in the upper atmosphere, producing fast neutrons that reach the Earth’s surface and penetrate the soil. Hydrogen atoms—primarily from water molecules—are highly effective at moderating these neutrons. As a result, the flux of returning fast neutrons to the atmosphere is inversely related to soil moisture content in the upper soil layers. CRNS sensors capture wide-area soil moisture averages, making them well-suited for field- and landscape-level monitoring. A sensor positioned ~ 2 m above ground typically senses moisture within a radius of approximately 130–240 m, covering a spatial footprint of several hectares, with most signal contribution coming from the upper tens of centimetres of soil. This large footprint effectively integrates surface heterogeneity due to varying topography, vegetation, and soil types, addressing the limitations of point-based sensors [55]. For example, the U.S. COSMOS network demonstrated regional-scale moisture monitoring over areas spanning tens of hectares. In another case within a ~ 1 km² watershed, a 24-station CRNS array was arranged to ensure overlapping footprints, enabling comprehensive monitoring across diverse terrain and vegetation zones [56, 57].

CRNS provides non-destructive, continuous soil moisture monitoring, bridging the spatial scale between point measurements and satellite remote sensing. According to IAEA, CRNS can cover areas on the order of tens of hectares, offering a complementary scale for soil moisture estimation. The above-ground installation requires minimal site disturbance compared to in-ground sensors, making CRNS an ideal choice for open-field applications, such as grassland and agricultural fields. Its broad horizontal footprint averages out small-scale variability, supporting robust detection of field-average soil moisture changes, including post-rain recovery or drought responses [56, 58]. CRNS fills the gap between point sensors and remote sensing by providing field-average, shallow-depth moisture dynamics that guide down-scaling and data assimilation.

In terms of limitations and adoption barriers, CRNS by definition yields an area-average value and lacks the ability to resolve fine spatial or vertical moisture variations. It is generally sensitive to the top 20–50 cm of soil, rarely exceeding ~ 80 cm in depth. Additionally, the method can be confounded by external hydrogen sources—such as live or dead biomass, surface water films, or snow cover—which affect neutron moderation and must be corrected in data processing. These calibration and correction requirements add operational overhead and can constrain adoption for plot-scale decision needs [59]. Practical uptake typically involves site-specific N₀ calibration accompanied by biomass/snow/water-film surveys, co-location with depth-profiling sensors to constrain vertical structure and guide down-scaling, and routine reporting of footprint, effective depth, and uncertainty, optionally complemented by periodic roving transects to characterize sub-field heterogeneity.

Recent innovations include mobile or “backpack” CRNS units that enable soil moisture mapping over large areas without fixed installations. These systems, suitable for mounting on vehicles or carried by personnel, can cover on the order of 20 ha per survey while maintaining non-invasive operation. “Roving” CRNS approaches, which combine mobile measurements with calibration sampling, have shown promise for mapping moisture variability across tens to hundreds of hectares in grassland and cropland environments [60].

### Graphene quantum-dot (GQD) sensors for moisture and ions

Graphene quantum dots are nanometer-scale fragments of graphene that retain high conductivity and surface area. Researchers have incorporated GQDs into resistive soil sensors. For instance, Kalita et al. (2016) fabricated a MEMS-based soil moisture sensor with interdigitated microelectrodes bridged by a film of GQDs [62]. When wet soil was placed on the sensor, its conductance changed dramatically with water content, from very low in dry soil to much higher in saturated soil. Notably, the GQD-sensor responded within ~ 2–3 min to moisture changes – reported as the fastest response among resistive soil-moisture devices. This high sensitivity and rapid response are attributed to the GQDs’ large surface area and ion-conducting pathways. GQD devices could, in principle, be tuned (via surface functionalization) to detect specific ions or pH in the soil solution. At plant-relevant scales, GQD devices offer miniaturized local readouts that can complement structural/spectral mapping by resolving near-surface heterogeneity once ruggedized and standardized.

GQD-based sensors combine low cost (graphene is abundant) and ease of fabrication with excellent electrical sensitivity. Another report shows the device with very large conductance shifts over 4–90% moisture and a very quick response (2–3 min) [62]. Such nanomaterial films amplify subtle chemical changes into measurable electrical signals. In practice, a single GQD sensor could be multiplexed for soil moisture, ionic strength, or pH by choosing appropriate coatings.

These sensors are still at the experimental stage and have undergone limited multi-season field validation, highlighting key limitations and barriers to adoption. The long-term stability of GQD films in soil, their susceptibility to fouling (organics/minerals), and temperature-/humidity-induced drift complicate calibration in complex media. The lack of standardized, cross-site calibration/QA procedures can also affect the comparability of readings. To date, there are few demonstrations beyond moisture; adapting GQD sensors to specific nutrients or pH in situ will require careful engineering of selectivity and anti-fouling power/communications integration, and maintenance plans for networked operation [63]. Nevertheless, if packaged robustly and supported by standardized calibration with multi-season field trials, GQD sensors could provide real-time high-resolution maps of soil moisture or nutrient gradients, offering early warning of drought stress or nutrient depletion before visual symptoms appear. Fielding GQD devices in the near term generally entails multi-season trials across contrasting soils, standardized calibration/QA with temperature compensation and anti-fouling measures, and rugged packaging with power/communications integration for networked operation. The initial emphasis is on moisture sensing before extending to ions or pH.

Taken together, emerging quantum-sensor approaches offer valuable and complementary views on soil–plant water status, yet practical constraints bound adoption. Neutron probes provide accurate profiles but remain point-scale and regulated due to sealed sources and operator requirements. CRNS delivers hectometer-scale area averages with shallow effective depth and demands site-specific calibration and corrections for external hydrogen pools (e.g., biomass, snow, surface films). GQD devices are pre-commercial, with outstanding questions on long-term stability, fouling, drift, and standardized calibration before routine field deployment. Cross-cutting priorities are (i) TRL-aligned validation in relevant environments, (ii) standardized calibration/QA and cross-site intercomparisons, (iii) ruggedization and power/communications integration for networks, (iv) operator training and maintainability, and (v) transparent cost and data-integration pathways with in-situ profiles and remote sensing. These steps will help clarify where each modality complements rather than replaces existing methods in grassland monitoring. Across quantum modalities, immediate priorities are multi-season validation in relevant environments, shared calibration/correction protocols, ruggedization and network integration, and explicit reporting of footprint, depth sensitivity, and uncertainty to enable sensor fusion and model–data assimilation.

### Best practices

Best practices in grassland deployments emphasize integrated use of quantum-based sensors. Field-average CRNS performs best when paired with depth-profiling instruments (e.g., neutron/TDR/FDR) and supported by site-specific calibration that accounts for biomass, snow, and surface water, with clear reporting of footprint, effective depth, and uncertainty to enable scaling and data assimilation [64].

#### Example 1

In a ~ 1 km² grassland headwater catchment, a dense CRNS array with overlapping footprints, periodic roving transects, co-located profile sensors, and biomass surveys provided stable field-average moisture dynamics and improved satellite downscaling [56, 64].

#### Example 2

At COSMOS-UK grassland sites, CRNS practice combines site-specific N₀ calibration, depth weighting for shallow sensitivity, and supplementary soil sampling for lattice/bound water. Co-located profile sensors are used for validation, along withetadata on footprint, depth, and corrections—approaches that improved seasonal tracking and eased dataset integration [57].

### Proximal and physiological sensors

#### Thermal sensor

Thermal sensors offer unique information for grassland monitoring that other sensors, such as multispectral or hyperspectral sensors, cannot provide. They can detect temperature changes on the Earth’s surface, indicating changes in soil moisture, vegetation health, and microclimate conditions. Thermal sensors can also provide spatial information on soil moisture and temperature, which can help optimize irrigation and management practices to improve crop yield and reduce water use. For example, evapotranspiration is also measured by the Landsat thermal sensor in grassland [65]. Thermal sensors can be used in combination with other sensors for a more comprehensive understanding of grassland ecosystems.

In contrast, thermal sensors in grassland research may have some drawbacks, such as limited spatial resolution compared to hyperspectral or multispectral sensors, restricted spectral information on vegetation properties, reliance on environmental conditions, high variability, and infrequent data collection, leading to limited temporal coverage.

#### Electrochemical sensor

Electrochemical sensors can be used in grassland research for various applications, such as monitoring nutrient levels in soil, assessing the health of plant roots, and tracking the concentrations of multiple gases in the atmosphere. Electrochemical sensors measure a sample’s electrical properties, such as the voltage or current produced by a chemical reaction. One example of electrochemical sensors in grassland research is a study by [66] which used a micro-electrochemical sensor for N_2_O monitoring in soil samples. This approach was already created in 1999, as reported by [42], to create an on-the-go automated soil monitoring system. The researchers found that the nitrate concentration varied significantly across different locations within the grassland. The highest concentrations were found near the edges of the ecosystem, where runoff from agricultural activities was more likely to occur.

Some of the strengths of electrochemical sensors in grassland research include their high sensitivity, ability to provide real-time measurements, and ability to be used in the field. However, some weaknesses include the need for calibration and maintenance, as well as the potential for interference from other chemicals in the sample. Also, it is reported that another bottleneck of using electrochemical sensors for soil monitoring is the change in nutrient contents over small spatial scales [67]. This can increase uncertainties of interpolative prediction, particularly when the sample volume is limited. An alternative approach is suggested for soil sampling, such as an “in-situ sensor” of ion-selective electrodes (ISEs), which easily calculates the concentration of the target ion via a pre-calibration (e.g., NO_3_ and NH_4_) [68, 69]. Compared to the electrochemical sensor, this approach is simple and relatively cheap, and an electrical power supply is not required.

#### Ultrasonic sensor

Ultrasonic sensors have been utilized in grassland research to measure vegetation structure, biomass, and productivity. Ultrasonic sensors emit high-frequency sound waves that reflect off vegetation and other objects, enabling researchers to estimate vegetation height, density, and cover [70]. They offer advantages over traditional field methods with faster data collection, higher accuracy, and non-destructive measurements (Fig. 6).

However, ultrasonic sensors also have limitations, such as a specific height range, which can be challenging in grassland research where vegetation height varies greatly, and the sensor may not detect tall vegetation. Environmental factors like wind, temperature, and humidity can also impact sensor accuracy and precision. Ultrasonic sensors can be expensive to purchase and maintain, may require specialized software for data processing, and can be affected by interference from other objects in the environment.

**Fig. 7:**
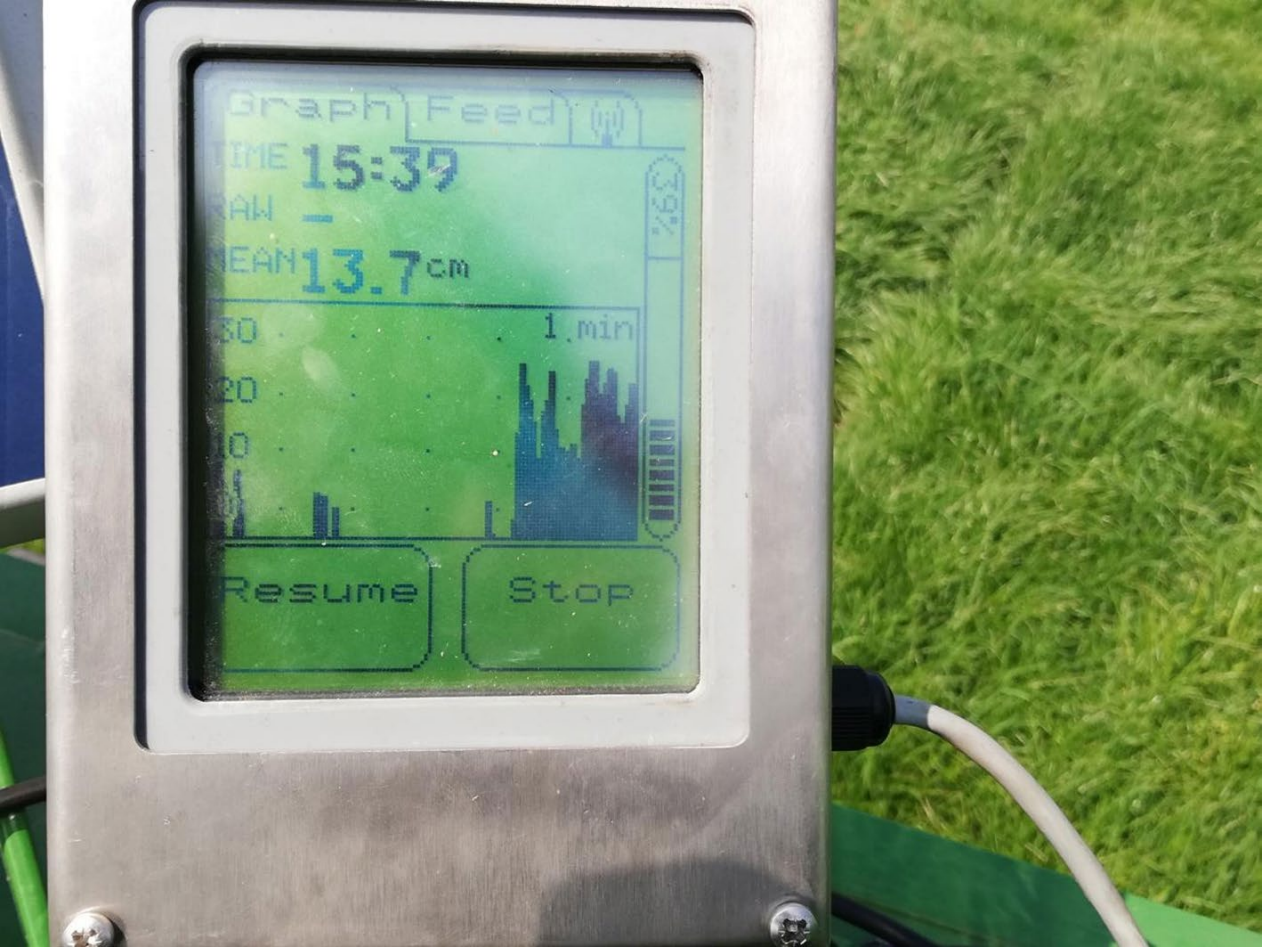
Ultrasonic sensor, Pasture Reader, for plant height measurement. http://pasturereader.com.au/

Ultrasonic sensor accuracy can be improved by combining with spectral sensors for biomass estimation. ML algorithms can filter environmental noise and correct for canopy irregularities (Fig. 7).

Despite these limitations, ultrasonic sensors are a valuable tool for grassland research. Combining with additional sensors can reinforce the accuracy of the measurement, such as biomass estimation [71].

#### LAI portable Anlayzer

The LAI Plant Canopy Analyzer, such as the LAI-2200 C (LI-COR^®^), is an optical sensor widely used in grassland research to estimate canopy structure, assess light interception, and evaluate plant productivity [72, 73]. The device operates by measuring the attenuation of diffuse sky radiation at multiple angles using a fisheye optical sensor. By comparing light intensities above and below the canopy, the system calculates the LAI using the gap fraction method, which assumes a probabilistic distribution of foliage. This non-destructive approach enables efficient monitoring of seasonal and environmental changes in grassland.

However, the analyzer has several disadvantages. It assumes a random foliage distribution, which may not accurately represent the clumped or heterogeneous canopy structure often found in grassland [74]. To address assumptions of uniform foliage distribution, LAI analyzers can be complemented with 3D scanning or UAV-based imaging. ML-based gap fraction models can improve accuracy under heterogeneous canopy conditions. Indirect LAI measurements can be influenced by background light conditions, sensor positioning, and the presence of understory vegetation, potentially leading to measurement errors [74]. To avoid this bias, researchers usually take measurements near sunrise or sunset to ensure nearly uniform sky illumination [72].

### Integration strategies and their challenges

Using multiple sensor types in grassland research is beneficial because it allows for more precise monitoring than relying on a single platform, such as satellites or UAVs [75, 76]. Integrating multiple sensor types in grassland monitoring remains challenging due to differences in spatial and temporal resolution, calibration, and data formats, which limit interoperability. Data fusion can improve accuracy—for example, combining SAR with optical data or CRNS with UAV imagery—but it also increases computational demands and requires specialized expertise. Machine learning methods offer potential solutions [77], yet they, too, involve significant costs and time.

From a practical perspective, the added value of integration must be balanced against financial and technical investments, particularly at the farm scale.

In addition to technical capabilities, the adoption of sensor technologies in grassland research depends on cost-effectiveness and return on investment. Table 2 summarizes typical costs, value potential, and practical challenges across major sensor categories, along with emerging developments. While multispectral and proximal sensors are relatively low-cost and widely scalable, more advanced modalities such as hyperspectral, LiDAR, and quantum sensors require greater investment but offer unique data streams. Cost–benefit considerations are therefore central when deciding which technologies to deploy at farm, regional, or research scale.

While individual sensors provide valuable information, their full potential emerges when integrated with modeling approaches that link observation with process understanding. The next section discusses how data assimilation connects sensor data with crop and grass growth models.

**Table 2:** Cost-effectiveness and practical considerations of sensor technologies for grassland monitoring

Sensor category	Typical costs & scalability	Value / ROI potential	Technical & practical challenges	Emerging developments	Example references
Structural sensors (e.g., LiDAR, GNSS-IR)	LiDAR instruments are costly, though UAV-mounted units are becoming more accessible. GNSS-IR is relatively low-cost.	High ROI for canopy height, biomass, and land-cover mapping.	UAV-LiDAR has limited temporal coverage; GNSS-IR requires calibration and has coarse resolution.	Miniaturized UAV LiDAR; integration with spectral data.	Moeckel et al., 2017 [44]; Zhang et al., 2018 [26]
Spectral sensors (multispectral, hyperspectral, NIRS, SIF)	Multispectral is low-cost and widely used; hyperspectral and SIF remain expensive.	Strong ROI for forage quality, vegetation indices, and species detection.	Hyperspectral data are large and costly; sensitive to clouds and atmosphere.	Cheaper UAV multispectral payloads; portable NIRS for on-farm feed quality.	Wang et al., 2019 [30]; Nishikawa et al., 2023 [43]; Parrini et al., 2022 [33]
Quantum sensors (neutron probes, CRNS, graphene quantum dots)	Neutron probes are costly and regulated; CRNS mid–high cost but covers large footprints; GQD sensors experimental.	High value for soil–water monitoring and drought management.	Safety regulation (neutron probes); coarse footprint (CRNS); calibration issues (GQD).	Mobile CRNS systems; printed/flexible graphene sensors.	Sun et al., 2023 [53]; Borsato et al., 2019 [60]; Kalita et al., 2016 [61]
Proximal & physiological sensors (thermal, electrochemical, ultrasonic, LAI analyzers)	Generally low–mid cost; many handheld or tractor-mounted devices.	Favorable ROI for field-scale stress, nutrient, and forage monitoring.	Limited coverage; environmental interference (e.g., wind, light).	IoT-enabled low-cost sensor networks; integration with UAV/RS platforms.	García-Santos et al., 2022 [38]; Kim et al., 2021 [39]; Moeckel et al., 2017 [44]

#### Grass growth model and its combination with RS technologies

Crop simulation models (CSMs) enable the prediction of the effects of agro-management decisions on crop variables by describing dynamic interactions. There are various CSMs available, ranging from simple ones that focus solely on crop water use (e.g., FAO-WRSI) to comprehensive models that simulate crop growth, phenology, photosynthesis, the growth of plant organs, and water/nitrogen use (e.g., DSSAT, WOFOST, APSIM). Plant functional-structural models take it further by explicitly describing growth and development at the individual plant level, including branch and leaf geometry, as well as competition for resources [78].

The grass growth model has been used worldwide for decades [79–85]. Originally, estimating daily potential grass growth rate was a composite of two functions (one relating grass species’ maximum growth rate to temperature and another relating daily temperature to time).

As time passes, more parameters are inserted to reinforce the growth rate prediction. The daily potential growth rate was adjusted using environmental temperature, leaf area, soil moisture, and nitrogen rate [79].

According to a previous review paper focusing on crop models [86], various grass growth models exist, depending on the objectives. For example, other researchers [86] conducted a comparative study of the models BASGR, CATIMO, and STICS for predicting crude protein (CP) concentration, neutral detergent fibre (NDF) concentration, and NDF digestibility in timothy grass (*Phleum pratense* L.). As a continuous study, they worked on additional targets such as greenhouse gas balance [87]. In contrast, other researchers [88] presented the CoSMO (Community Simulation Model) for the dynamic simulation of the relative abundance of grass species.

Despite their potential in understanding crop growth, weather, and management interactions, the application of CSMs in precision agriculture is often hindered by uncertainties. These uncertainties can reduce the accuracy of the model’s predictions, diminishing the benefits of using a CSM. In precision agriculture, uncertainties are often related to poorly simulated model processes due to a lack of knowledge, intra-field variation in soil properties, or unaccounted factors such as pests and diseases. Additionally, factors that are difficult to describe accurately, such as local frost kill caused by varying snow depth due to micro-relief, also add to the uncertainties.

Using the data assimilation approach can decrease uncertainties in CSMs by incorporating external observations of crop variables into the model.

Data assimilation of RS data into the crop growth model can be applied to modify the model for the impact of factors not accounted for in the model structure (biophysics) or for uncertainty about certain model inputs. Essentially, DA can help resolve discrepancies between model predictions and real-world observations.

Regarding grassland research, several reports exist on this approach for grassland monitoring [89–91]. He et al. [54] reported integrating the LAI value from MODIS into the Soil-water-atmosphere-plant (SWAP) model. They improved R^2^ from 0.73 to 0.76 for biomass estimation after applying a four-dimensional variational data assimilation algorithm (4D-VAR). Other researchers [30] showed the integration of RS data into the PILOT model, which simulates vegetation growth for hay crops. LAI values and harvest dates were estimated from optical images (Landsat 7/8, Spot 4/5, and Sentinel 2A/2B).

In contrast, irrigation dates are inferred from SAR (Synthetic Aperture Radar) data from Sentinel 1, COSMO-Skymed, and TerraSAR-X. This study focused on the effects of uncertain or missed irrigation dates, i.e., poorly known agricultural water management options, on model predictions. Other researchers [89] used the data from MODIS and Landsat in the WOFOST model. They applied the Kalman filter to correct the LAI simulation by inputting the observed LAI. Huang et al. [58] tested the DA approach in data-limited areas, which occur more frequently in grasslands compared to cereal crop fields. They demonstrated the incorporation of MODIS data (LAI, GPP, and ET) into the BASGRA model using the Ensemble Kalman Filter and Bayesian calibration methods. As a result, even in a small number of field measurements, the predictive accuracy in the model has been improved.

For future studies in the DA approach, additional computing reversing schemes should be explored to update state variables other than LAI, which is commonly used in the DA approach [91]. Also, the reinforcement of the quality of input RS data should be targeted before the assimilation into the CGM [70, 91].

In addition, recent progress in UAV-based and proximal sensing offers valuable opportunities to enhance DA frameworks. For example, Parra et al. (2024) [92] demonstrated that drones equipped with multispectral and thermal sensors can map fine-scale spatial variability and stress responses in turfgrass systems, providing dense, high-frequency data that complement satellite observations. Similarly, Cantu (2024) [93] employed small unmanned aerial systems (sUAS) with dual NIR bands (850 nm and 970 nm) to detect drought stress in golf-course fairways, showing that localized stress detection can inform model correction and parameter updating.

Integrating such high-resolution UAV observations into DA workflows could help bridge the scale gap between satellite-based inputs and field-scale crop models. In particular, these UAV datasets could serve as intermediate assimilation layers—validating or constraining soil moisture, canopy temperature, or stress-related parameters—and thereby improve the reliability and responsiveness of crop and grassland growth models under heterogeneous management and climatic conditions.

As data assimilation improves the accuracy of biophysical predictions, digital platforms and decision-support systems play a crucial role in translating these insights into practical management actions.

#### Digital platform and decision-support service

Digital platforms can greatly assist farmers, extension officers, researchers, agribusiness companies, and policymakers in managing grassland ecosystems. Digital platforms offer real-time data on grassland conditions like soil health, moisture levels, and vegetation cover. These data help farmers and extension officers make informed decisions regarding grazing management, fertilization, and other practices that can improve the productivity and health of grassland. Policymakers can also benefit from these platforms as they provide information at regional and national scales, which can inform policy decisions related to land use, conservation, and resource management. Some examples of digital platforms for grassland management include SatSure, FarmBeats, and AgriSat. These platforms leverage satellite imagery and other data sources to provide comprehensive information on grassland conditions. By facilitating knowledge sharing and collaboration among stakeholders, digital platforms have the potential to revolutionize grassland management and support these critical ecosystems.

Sen2Grass is a digital open-source platform focusing on grassland fields created for an automated monitoring system [94]. Sentinel 1 and 2 are used with different machine-learning algorithms to detect mowing events.

Not only in the private sector, where companies and startups lead the development of digital platforms, but also in the public sector, national and international programs utilize remote sensing data for grassland monitoring. As an example, POLWET, a project funded by the European Space Agency, focuses on wetland monitoring in Poland’s Ramsar Convention sites where grassland areas are dominant. It integrates multi-temporal and high-resolution satellite data, including Landsat, Sentinel, MODIS, and Envisat radar images, to track land cover changes, water surface dynamics, moisture conditions, and biomass development [32]. The project provides maps and vegetation indices, including grassland areas that support environmental monitoring and conservation efforts. By developing an Earth observation (EO)-based information service, POLWET enhances accessibility to key data, which can contribute to broader international initiatives such as GlobWetland [95].

In Northern Ireland, the GRASSCHECK project was established to assist farmers with grassland management decisions. It was organized by AgriSearch, a non-profit organization that operates as a publicly funded research body, in collaboration with the Agri-Food and Biosciences Institute (AFBI). This service has now expanded to a UK-wide scale, providing continuous weekly grass growth predictions. In France, a 10 m map of natural grassland on the mainland was created using Sentinel 2 data, in conjunction with long-term observations and input from stakeholders in ecology, agriculture, and land-use planning [96].

FarmMaps is another digital platform in The Netherlands that provides farmers and land managers with detailed mapping and analysis tools on a global scale to improve their land management practices. Using satellite imagery and other data sources, FarmMaps generates high-resolution maps of farmland that can help farmers identify areas that need attention. Several applications support farmers, such as site-specific fertilizer application, pest and disease management, and soil moisture monitoring. In grassland management, the platform includes an application that measures grass height and estimates the feed wedge, representing the ranked grass yield available to dairy farmers [97]. Another application related to grassland is the “WatBalSig” application, a dynamic model for predicting soil moisture content. This model encompasses several factors, including evapotranspiration, precipitation, irrigation, and drainage [97]. By providing a “grassland calendar”, FarmMaps aims to optimise land management, reduce waste, improve productivity, and identify opportunities for sustainable land use. The NASA Applied Remote Sensing Training (ARSET) program offers a course on monitoring invasive species in grassland using remote sensing. This training provides an overview of NASA satellites and sensors utilized to map invasive plants, including Landsat, MODIS, and VIIRS [98].

Together, these advances—from sensing and modeling to decision delivery—illustrate an emerging continuum in grassland monitoring.

## Conclusion

Sensor technologies have advanced significantly in grassland research over recent decades, providing powerful tools for monitoring vegetation, soil, and climate dynamics. Structural and spectral sensors, alongside emerging quantum technologies, offer complementary perspectives, while digital platforms increasingly facilitate access and usability for farmers, researchers, and policymakers.

Recent studies show a growing reliance on data-driven approaches. Machine learning and deep learning methods (e.g., RF, SVM, and convolutional neural networks) are now used to classify and predict grassland traits, while data fusion techniques (e.g., SAR + optical, UAV + CRNS) improve monitoring accuracy despite challenges with calibration, interoperability, and computational cost.

Despite rapid advances, current sensor applications in grasslands face practical constraints: limited or uneven spatial/temporal coverage (e.g., UAV LiDAR, thermal), heavy calibration and correction requirements (NIRS, GNSS-IR, CRNS), environmental sensitivity (optical, thermal, ultrasonic), and unresolved long-term stability for emerging nanomaterial devices (GQDs). Point-scale (neutron probe) versus area-average (CRNS) versus pixel-based (optical/SAR) outputs create scaling challenges, while data fusion and ML increase computational and expertise demands. Interoperability and standardization remain barriers, alongside costs, regulatory requirements, and the need for multi-season, cross-site validation and shared QA/QC protocols.

Looking ahead, three priorities emerge. First, future research should strengthen sensor integration and model–data assimilation, linking remote and proximal sensing with crop growth and ecosystem models. Second, efforts are needed to address interoperability and standardization, including the adoption of digital communication protocols (e.g., ISOBUS) to connect sensors with decision-support platforms. Third, there is a significant opportunity in stakeholder-oriented applications, such as linking livestock dietary monitoring with grass quality assessment, evaluating ecosystem services, and assessing environmental impacts such as nutrient leaching. By combining advances in sensor hardware, data fusion, and artificial intelligence, grassland research can move toward more precise, scalable, and actionable insights—ultimately supporting both sustainable land management and climate resilience.

### Future directions in sensor-based grassland research

- *Advanced data analytics* Apply machine learning and deep learning (e.g., random forest, SVM, CNNs) to improve classification, prediction, and synthesis of multi-sensor data.
- *Data fusion and integration* Combine complementary sensors (e.g., SAR + optical, UAV + CRNS) and link them with crop simulation and ecosystem models.
- *Interoperability and standardization* Develop protocols (e.g., ISOBUS) to ensure compatibility across platforms and enhance digital decision-support systems.
- *Stakeholder-oriented applications* Connect livestock monitoring with grass quality, integrate environmental impact assessment (e.g., nutrient leaching), and design tools aligned with farmer and policymaker needs.
- *Emerging technologies* Explore novel approaches such as quantum sensors and edge-computing platforms for real-time, high-sensitivity monitoring.

## Supplementary Information

Supplementary Material 1.

## Data Availability

not applicable.

## Abbreviations

CRNS: Cosmic-ray neutron sensing (Field-scale soil moisture monitoring)
DA: Data assimilation (Integration of sensor data into grass/crop growth models)
GNSS-IR: Global navigation satellite system interferometric reflectometry (Measures vegetation height, biomass, soil moisture)
LAI: Leaf area index (Indicator of canopy density and productivity)
LiDAR: Light detection and ranging (Structural canopy and terrain mapping)
ML: Machine learning (Data fusion, classification, predictive modeling)
NIRS: Near-infrared spectroscopy (Forage quality, species identification, soil composition)
RF: Random Forest (Used for robust classification and biomass estimation from sensors)
RS: Remote sensing (General term for satellite- or UAV-based monitoring)
SAR: Synthetic aperture radar (Active microwave sensing; soil moisture, mowing, disturbance detection)
SIF: Sun-induced fluorescence (Proxy for photosynthetic efficiency)
SVM: Support vector machines (Application for accurate vegetation classification with limited training data)
UAV: Unmanned aerial vehicle (Platform for high-resolution proximal sensing)
