# Supplementary material for: Unlocking the synergistic potential of sensor technologies in grassland research
Source: Discov Sens. 2025 Nov 17;1(1):19. doi: 10.1007/s44397-025-00020-2 (PMC12645823; doi:10.1007/s44397-025-00020-2)
Supplement: Supplementary file 1 — Supplementary Material 1. [file 44397_2025_20_MOESM1_ESM.docx]

**Supplementary material**

We conducted a literature survey and employed bibliometric analysis to gain a comprehensive understanding of sensor technologies in grassland research. Bibliometrics is a statistical approach that quantitatively analyzes research papers on a specific topic using mathematical methods. VOSviewer (Van Eck and Waltman, 2010) is a continuously evolving application used for this purpose. Our data collection began with Scopus, using the keyword "sensor AND grassland" with a title filter. Publications from 1977 to 2023 were considered, resulting in a total of 919 papers. After exporting this paper collection in CSV format, we imported it into the VOSviewer program. We selected "co-occurrence" as the analysis type, opting for overlay visualization for the final representation to show the chronological change over the last ten years (from 2012 up to now). This analysis was conducted on March 8, 2023, as part of our research project.

van Eck, N.J., Waltman, L. (2010). Software survey: VOSviewer, a computer program for bibliometric mapping. Scientometrics, 84, 523–538. https://doi.org/10.1007/s11192-009-0146-3
